# Identification and Evaluation of Menstruation-Related Disorders in Adolescent Girls Attending a Tertiary Care Center: An Observational Study

**DOI:** 10.7759/cureus.99192

**Published:** 2025-12-14

**Authors:** Pragya Prem, Veena S R, Meena T S, Revathy T.G

**Affiliations:** 1 Obstetrics and Gynaecology, Sree Balaji Medical College and Hospital, Chennai, IND

**Keywords:** adolescent health, anemia, dysmenorrhea, india, menorrhagia, menstrual hygiene, premenstrual syndrome, school absenteeism, whodas

## Abstract

Background

Menstrual disorders such as dysmenorrhea, menorrhagia, oligomenorrhea, and premenstrual syndrome are believed to be the most common yet under-recognized causes of morbidity in adolescent girls. Many adolescents around the world experience menstrual dysfunction, and in India, cultural stigma, poor awareness, and nutritional imbalance are believed to exacerbate the burden. Despite national initiatives focusing on menstrual hygiene and anemia prevention, only a few studies in India have systematically evaluated the clinical spectrum, determinants, and functional impact of menstrual disorders in tertiary-care settings. This study aimed to assess the prevalence, pattern, and clinical correlates of menstruation-related disorders among adolescent girls attending a teaching hospital in South India and to identify the functional limitations imposed by these conditions.

Methods

A hospital-based cross-sectional observational study was conducted among 72 post-menarcheal adolescent girls (10-19 years) attending the outpatient department of Obstetrics and Gynecology, Sree Balaji Medical College and Hospital, Chennai. Participants were assessed using a validated, semi-structured questionnaire covering sociodemographic data, menstrual characteristics, hygiene practices, and psychosocial impact. Clinical evaluation included anthropometry, hemoglobin estimation, thyroid function testing, and pelvic ultrasonography where indicated. The severity of symptoms and functional disability were assessed using the Pictorial Blood Assessment Chart (PBAC), Visual Analogue Scale (VAS), and WHO Disability Assessment Schedule (WHODAS 2.0).

Results

The mean age at menarche was 12.6 ± 1.2 years, with 69.4% reporting regular cycles and a mean duration of flow of 4.9 ± 1.6 days. The overall prevalence of menstrual disorders was strikingly high (94.4%). Dysmenorrhea (72.2%) and premenstrual syndrome (75.0%) were the most common, followed by menorrhagia (25%) and oligomenorrhea (13.9%). Anemia was observed in 33.3%, thyroid abnormalities in 8.3%, and polycystic ovary syndrome (PCOS) features in 13.9% on ultrasonography. Overweight and obesity were significantly associated with menstrual irregularities (p=0.013). Functionally, participants missed an average of 1.8 ± 0.9 school days per cycle, with 38.9% reporting absenteeism and 47.2% restricting social or physical activities. The mean WHODAS disability score was 8.6 ± 3.5, showing a strong correlation (r=0.62; p<0.001) with menstrual symptom severity.

Conclusion

Menstrual disorders are highly prevalent among adolescents and impose significant physical and psychosocial burdens. Integrating menstrual health education, nutritional interventions, and early clinical screening into adolescent programs is vital to improving reproductive health and educational outcomes.

## Introduction

Adolescence represents a dynamic stage of human development marked by rapid physical growth, hormonal transition, and psychological adaptation. One of the most important physiological milestones during this period is the onset of menstruation, or menarche, which signifies the maturation of the hypothalamic-pituitary-ovarian (HPO) axis and the attainment of reproductive potential [1]. The menstrual cycle is a finely regulated process dependent on pulsatile secretion of gonadotropin-releasing hormone (GnRH) from the hypothalamus, coordinated release of gonadotropins from the anterior pituitary, and cyclic ovarian steroid feedback. Disruption of any component of this pathway can lead to menstrual irregularities. During the early years after menarche, the immaturity of the HPO axis commonly results in anovulatory and irregular cycles that may persist for one to two years [1]. Menarche generally occurs two to three years after thelarche and is influenced by genetics, nutrition, body composition, and environmental factors. While short-term irregularities may be physiological, persistence beyond two years or the presence of heavy or painful bleeding indicates menstrual dysfunction requiring evaluation [2].

Menstrual disorders encompass a broad range of abnormalities in cycle frequency, duration, and volume, including amenorrhea, oligomenorrhea, menorrhagia, polymenorrhea, dysmenorrhea, and premenstrual syndrome. These conditions collectively impose a substantial global burden on adolescent health. Worldwide studies report that between half and three-quarters of adolescent girls experience at least one form of menstrual disorder, with dysmenorrhea being the most frequent complaint [3-5]. A community-based study from Osogbo, southwestern Nigeria, showed that over two-thirds of adolescent girls suffered from menstrual problems, primarily dysmenorrhea and menorrhagia, which led to school absenteeism and reduced academic concentration [3]. The study further highlighted that misconceptions surrounding menstruation, social taboos, and lack of awareness prevented many from seeking medical care, resulting in chronic suffering and normalization of menstrual pain [3]. Similar patterns have been observed across East Africa, where a study conducted among undergraduate students at Makerere University in Uganda found a high prevalence of menstrual disorders that substantially affected educational performance and psychosocial well-being [4]. Participants frequently reported missing classes, emotional distress, and fatigue during menstruation, underscoring that menstrual disorders extend beyond biological discomfort to broader social and functional impairment [4].

A large questionnaire-based study in North Borneo, Malaysia, revealed that about 64.5% of adolescent girls experienced at least one menstrual disorder, with premenstrual syndrome and dysmenorrhea being the most common [5]. The study identified rural residence, limited menstrual-health knowledge, and low socioeconomic status as major risk factors. These findings indicate that the burden of menstrual morbidity is amplified by inadequate education and limited access to sanitary products, particularly in developing regions [5].

Within the Indian context, menstrual disorders represent one of the most common yet under-recognized health problems among adolescents. India has nearly 253 million adolescents, forming a critical segment of the national population, and menstrual health directly influences their physical, mental, and academic development. A school-based study conducted in rural Tamil Nadu demonstrated that 75% of adolescent girls experienced dysmenorrhea, 23% reported menorrhagia, and 12% had irregular cycles [6]. Nutritional status and body mass index were found to have significant associations with menstrual irregularities. The authors reported that most adolescents relied on home remedies or endured symptoms silently due to stigma and lack of reproductive-health awareness [6]. These findings illustrate a consistent pattern of high prevalence and poor help-seeking behavior, which continues to challenge public-health interventions in India. Despite national programs such as the Rashtriya Kishor Swasthya Karyakram and School Health Program, menstrual health has not received proportional emphasis, and most initiatives remain focused on hygiene and anemia rather than cycle regularity or underlying pathology.

Menstrual problems have far-reaching consequences beyond physical discomfort. Studies have shown that adolescents with menstrual disorders have lower health-related quality-of-life (HRQoL) scores, higher anxiety, and greater emotional instability compared to unaffected peers. Pogodina et al. demonstrated that adolescents experiencing dysmenorrhea or heavy bleeding reported impaired school performance and reduced social participation [7]. The authors emphasized that untreated menstrual symptoms can lead to long-term absenteeism and psychological distress [7]. Even in high-income Asian countries, menstrual health challenges persist. In Singapore, a cross-sectional study among schoolgirls found that one-quarter had irregular cycles and more than half suffered from dysmenorrhea [8]. Despite the availability of medical services, adolescents rarely consulted healthcare professionals, citing embarrassment and normalization of pain as key barriers. The study underscored that societal silence surrounding menstruation continues to impede timely care, even in technologically advanced contexts [8].

Evidence from the Middle East further reinforces the global nature of this problem. A study among secondary-school girls in Egypt showed that over 70% experienced menstrual irregularities, while nearly half suffered premenstrual symptoms [9]. Stress, obesity, and poor dietary habits were significant predictors. The authors noted that urbanization, sedentary lifestyle, and changing dietary patterns have led to rising menstrual dysfunction in the adolescent population [9]. These findings highlight that rapid modernization, coupled with academic pressure and lifestyle transitions, may contribute to increasing menstrual problems in low- and middle-income nations undergoing socio-economic change.

Emerging research also shows that psychological stress and environmental instability have profound effects on menstrual regularity. During the ongoing conflict in Ukraine, a cross-sectional study among adolescent girls revealed a marked increase in stress-related menstrual disorders such as oligomenorrhea and secondary amenorrhea [10]. The authors attributed this to psychological trauma, displacement, and nutritional deficiencies. Their observations confirm that menstrual health is highly sensitive to both physical and emotional stressors. These findings are especially relevant to modern adolescents who face intense academic competition, social media pressures, and lifestyle disruptions-factors increasingly recognized as triggers for menstrual irregularities worldwide [10].

While school-based surveys have provided prevalence data, there is a paucity of hospital-based research evaluating the clinical spectrum of menstrual disorders, their etiological correlates, and their impact on adolescents’ well-being. Tertiary-care settings offer a unique opportunity for detailed clinical evaluation, including hormonal assessment, anthropometric profiling, and psychosocial analysis, that community studies cannot achieve. Such evidence is crucial to design targeted interventions, identify high-risk groups, and develop standardized protocols for diagnosis and management. Addressing these issues is therefore essential not only for improving reproductive health but also for achieving broader developmental goals linked to education, empowerment, and equity. This study aimed to assess the spectrum of menstruation-related disorders among adolescent girls attending a tertiary care center and to examine their clinical characteristics, associated determinants, and impact on daily functioning.

## Materials and methods

Study design, setting and duration

This hospital-based cross-sectional observational study was conducted in the Department of Obstetrics and Gynaecology at Sree Balaji Medical College and Hospital, Chennai, which serves a mixed urban and rural population in South India. The study was carried out over a period of two months, from September 2025 to October 2025, with the objective of identifying the prevalence, patterns, and determinants of menstruation-related disorders among adolescent girls.

Study population

The study population consisted of adolescent girls aged 10 to 19 years who had attained menarche and attended the outpatient department either for menstrual complaints or other gynecological issues during the study period.

Inclusion and exclusion criteria

Adolescent girls who had attained menarche and provided written informed consent or assent, with parental consent for minors, were included in the study. Those with congenital anomalies of the reproductive tract, girls undergoing hormonal therapy for non-menstrual causes, and those who were critically ill or unable to cooperate were excluded.

Sampling technique and sample size

Participants were selected through consecutive sampling until the desired sample size was achieved. The sample size was calculated using Dobson’s formula for a single population proportion, considering a prevalence of 50% and a precision of 11.7% at a 95% confidence interval. Based on this calculation, a minimum of 70 participants was required, and additional participants were included to account for possible non-response.

Procedure

Each participant was interviewed privately after obtaining informed consent, and confidentiality was maintained throughout the process. A pre-tested, semi-structured questionnaire in English and Tamil was used for data collection. A pre-tested, semi-structured questionnaire in English was translated into Tamil and back-translated into English by bilingual experts to ensure semantic equivalence. The questionnaire was subjected to content validation by a panel of three subject experts from the Departments of Obstetrics and Gynecology and Community Medicine, who assessed its relevance, clarity, and comprehensiveness. A pilot study was conducted among 10 adolescent girls with similar characteristics attending the same outpatient department who were not included in the final analysis. Based on feedback from the pilot testing, minor modifications were made to improve clarity and sequencing of questions. The finalized questionnaire was then used for data collection, which included sections on socio-demographic characteristics such as age, education, and socioeconomic status (classified using the Modified Kuppuswamy Scale, 2024). Details about specific menstrual disorders such as dysmenorrhea, menorrhagia, oligomenorrhea, polymenorrhea, and amenorrhea were recorded, along with associated symptoms like premenstrual tension, acne, and hirsutism.

Menstrual hygiene practices, including the type of absorbent used, frequency of change, and hygiene maintenance, were also documented. Participants were asked about the effect of menstruation on academic performance, school absenteeism, and social participation. Clinical examination included measurement of height and weight for body mass index (BMI) calculation and assessment for pallor, acne, hirsutism using the Ferriman-Gallwey score, and thyroid enlargement. Relevant investigations, such as hemoglobin estimation and thyroid function tests, were performed as clinically indicated. Pelvic ultrasonography was undertaken in participants presenting with abnormal uterine bleeding (PBAC score >100), secondary amenorrhea, severe or refractory dysmenorrhea, clinical features suggestive of polycystic ovary syndrome (such as oligomenorrhea with hirsutism or acne), suspected pelvic pathology on clinical examination, or when structural causes were considered based on history and examination findings. The intensity of menstrual pain was assessed using the Visual Analogue Scale (VAS), menstrual blood loss was quantified using the Pictorial Blood Assessment Chart (PBAC), and premenstrual symptoms were evaluated using the Premenstrual Symptoms Screening Tool (PSST). The functional impact of menstrual disorders on daily activities was assessed using the WHO Disability Assessment Schedule (WHODAS 2.0).

Menstrual disorders were defined according to standard clinical criteria. Dysmenorrhea was described as painful menstruation that limited daily activities. Menorrhagia was defined as menstrual blood loss exceeding 80 mL or bleeding lasting for more than seven days per cycle. Oligomenorrhea was defined as cycles occurring at intervals greater than 35 days, polymenorrhea as cycles shorter than 21 days, and amenorrhea as the absence of menstruation for three consecutive months in previously regular cycles. These definitions ensured uniformity in classification and comparison across participants.

Data analysis

All interviews and examinations were conducted by the investigator under the supervision of the guide to ensure accuracy and consistency. Data were recorded in standardized case record forms and subsequently entered into Microsoft Excel. Statistical analysis was performed using IBM Corp. Released 2019. IBM SPSS Statistics for Windows, Version 25. Armonk, NY: IBM Corp. Descriptive statistics such as mean, standard deviation, and percentage were used to summarize demographic characteristics and prevalence of various menstrual disorders. Inferential statistics, including the chi-square test and Fisher’s exact test, were used to assess associations between categorical variables, while Student’s t-test and the Mann-Whitney U test were applied for continuous variables depending on data normality. Binary logistic regression was performed to identify independent predictors of menstrual disorders after adjusting for confounding factors such as BMI, stress level, and socioeconomic class. A p-value of less than 0.05 was considered statistically significant.

Ethical consideration

Ethical approval for the study was obtained from the Institutional Human Ethics Committee of Sree Balaji Medical College and Hospital, Chennai. All participants and their guardians were informed about the study objectives, procedures, voluntary participation, and confidentiality safeguards. Written informed consent and assent were obtained prior to data collection. No experimental interventions were conducted, and all assessments were within the scope of routine clinical care. The study posed minimal risk to participants and ensured that refusal or withdrawal from participation did not affect their medical care in any way. Data confidentiality was strictly maintained through anonymization of participant identifiers, and records were stored securely with restricted access. The study did not receive any external funding or sponsorship and was entirely self-financed by the investigator.

## Results

A total of 72 postmenarchal adolescent girls were included in the present study. The findings are presented under sociodemographic characteristics, menstrual profile, prevalence of menstrual disorders, functional impact, and associated clinical parameters.

Table 1 shows the socio-demographic profile of the participants. The mean age at menarche was 12.6 ± 1.2 years, and the majority (n=35, 48.6%) were in the 14-16-year age group. About 69.4% (n=50) reported regular cycles with a mean duration of flow of 4.9 ± 1.6 days and a mean cycle length of 28.9 ± 4.5 days. One-fourth (n=18, 25%) had a Pictorial Blood Assessment Chart (PBAC) score ≥ 100, indicating heavy flow. Regarding clinical parameters, 25% (n=18) of participants were overweight or obese, 33.3% (n=24) were anemic (Hb < 12 g/dL), and 13.9% (n=10) showed polycystic ovarian morphology on ultrasonography.

**Table 1:** Baseline characteristics and menstrual profile of study participants (N=72) PCOS: polycystic ovary syndrome, BMI: body mass index, PBAC: pictorial blood assessment chart

Sociodemographic characteristics	Frequency
Age group	n (%)
10–13 years	12 (16.7)
14–16 years	35 (48.6)
17–19 years	25 (34.7)
Socioeconomic status	n (%)
Upper/Upper middle	18 (25.0)
Lower middle	30 (41.7)
Upper lower/Lower	24 (33.3)
Menstrual characteristics	Mean ± SD
Age at menarche (years)	12.6 ± 1.2
Cycle length (days)	28.9 ± 4.5
Duration of flow (days)	4.9 ± 1.6
Menstrual characteristics	n (%)
Regular cycles	50 (69.4)
PBAC score ≥100	18 (25.0)
Clinical and Laboratory Parameters	
BMI category	n (%)
Normal/Underweight	54 (75.0)
Overweight/Obese	18 (25.0)
Anemia (Hb <12 g/dL)	24 (33.3)
Abnormal thyroid profile	6 (8.3)
PCOS features on USG	10 (13.9)

Table 2 demonstrates the prevalence and severity of menstrual disorders among the study participants. Overall, 94.4% (n=68) of adolescents reported at least one menstrual disorder. Premenstrual syndrome (n=54, 75%) and dysmenorrhea (n=52, 72.2%) were the most prevalent conditions. These findings underscore the high burden of menstrual morbidity in this cohort.

**Table 2:** Prevalence and severity of menstrual disorder (N=72)

Menstrual Disorder	Frequency n (%)
Dysmenorrhea (n=52)	52 (72.2)
Premenstrual Syndrome (n=54)	54 (75.0)
Menorrhagia	18 (25.0)
Oligomenorrhea	10 (13.9)
Polymenorrhagia	6 (8.3)
Amenorrhea	3 (4.2)
Any menstrual disorder	68 (94.4)

**Figure 1:**
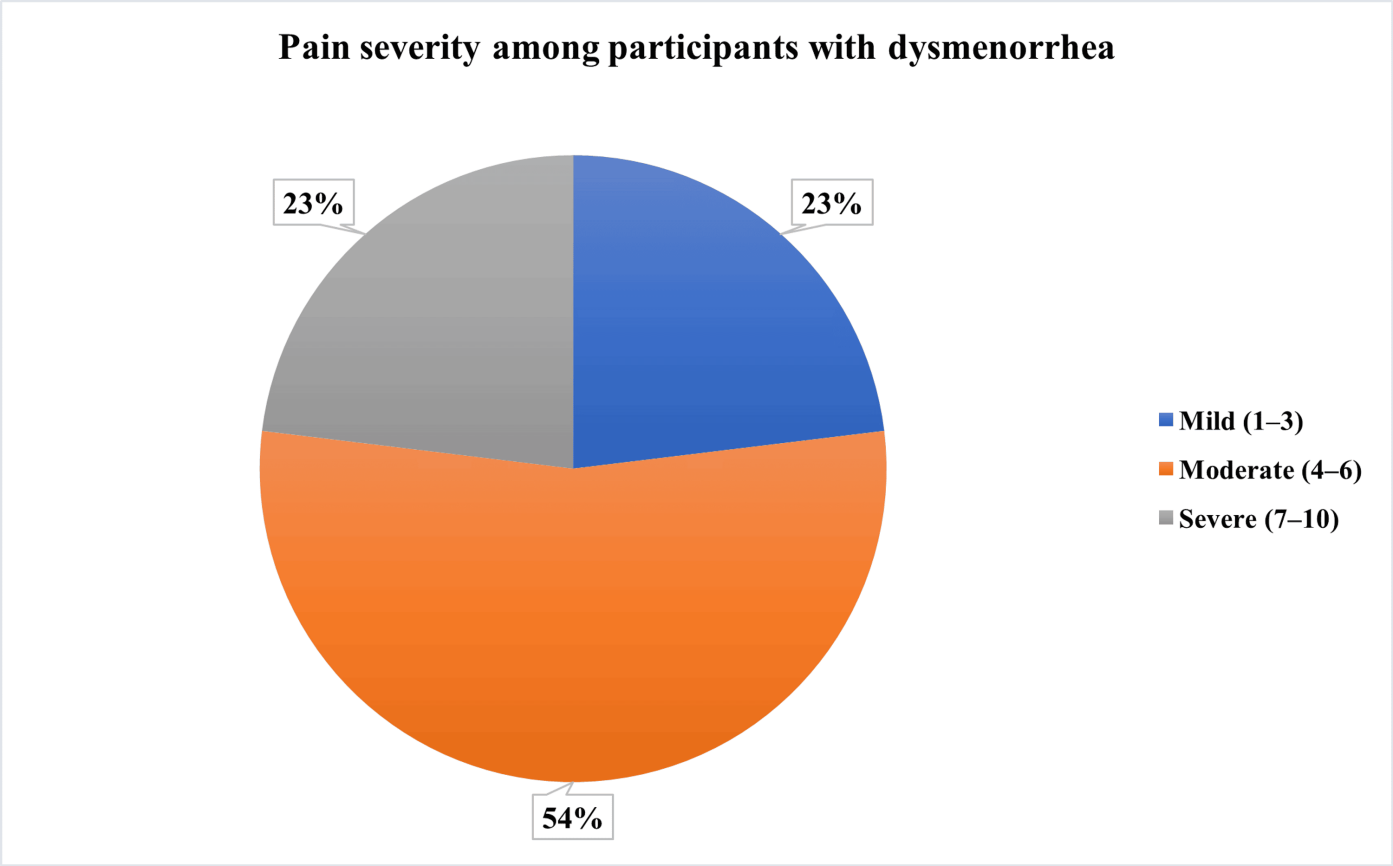
Pain severity among participants with dysmenorrhea (n=52)

Figure 1 illustrates the distribution of the severity of pain among the study participants. The majority of participants with dysmenorrhea reported moderate pain intensity (n=28, 54%) on the Visual Analogue Scale, followed by severe pain intensity (n=12, 23%).

**Figure 2:**
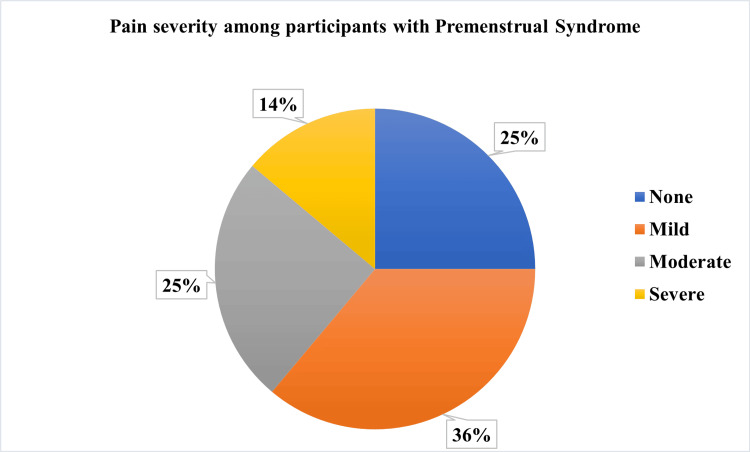
Pain severity among participants with premenstrual syndrome (n=54)

Figure 2 illustrates the distribution of the severity of pain among participants with premenstrual syndrome. Most reported moderate (n=13, 25%) physical and psychological symptoms, highlighting substantial discomfort prior to menstruation.

Table 3 demonstrates the functional impact of menstrual disorders on daily activities among the study participants. Menstrual morbidity significantly affected day-to-day functioning. Participants missed an average of 1.8 ± 0.9 school days per cycle. 38.9% (n=28) reported school absenteeism, and 47.2% (n=34) restricted social or physical activities during menstruation.

**Table 3:** Functional impact of menstrual disorders on daily activities (N=72) MSQ: Menstrual Symptom Questionnaire, WHODAS: World Health Organization Disability Assessment Schedule. Pearson correlation test used. p-value <0.05 – Statistically significant

Impact Domain	Mean ± SD
Days missed per cycle	1.8 ± 0.9
WHODAS disability score	8.6 ± 3.5
Total MSQ score	39.4 ± 9.8
Pain symptom	8.4 ± 3.2
Psychological symptom	7.5 ± 3.1
Autonomic symptom	6.7 ± 2.8
Behavioral/lifestyle impact	6.1 ± 2.6
Impact Domain	n (%)
School absenteeism	28 (38.9)
Restriction of social/physical activities	34 (47.2)
Correlation	
MSQ vs. WHODAS (r value, p-value)	0.62, <0.001

The mean World Health Organization Disability Assessment Schedule (WHODAS) score was 8.6 ± 3.5, and the mean Menstrual Symptom Questionnaire (MSQ) total score was 39.4 ± 9.8. There was a strong positive correlation between MSQ and WHODAS scores (r = 0.62, p < 0.001), indicating that higher symptom severity corresponded with greater functional disability.

Table 4 exhibits the association between clinical features and menstrual irregularities. Menstrual irregularity showed a significant relationship with body mass index and ovarian morphology. A) Overweight/obesity was significantly associated with menstrual irregularity (χ² = 6.3, p = 0.013). B) Presence of polycystic ovarian morphology on ultrasonography was also significantly associated (χ² = 7.3, p < 0.01).

**Table 4:** Association of clinical features with menstrual irregularities (N=72) BMI: Body mass index, PCOS: polycystic ovary syndrome Chi-square test used. p-value <0.05 – Statistically significant.

Category	Disorder Present (n = 50) n (%)	Disorder Absent (n=22) n (%)	χ2 value	p-value
BMI Status				
Normal/Underweight	42 (77.8)	12 (22.2)	6.3	0.013
Overweight/Obese	8 (44.4)	10 (55.6)		
Pelvic Ultrasound				
Normal	48 (75.0)	16 (25.0)	7.3	<0.01
PCOS features	2 (25.0)	6 (75.0)		

These findings highlight the metabolic and endocrine contributions to menstrual dysfunction in adolescents.

**Figure 3:**
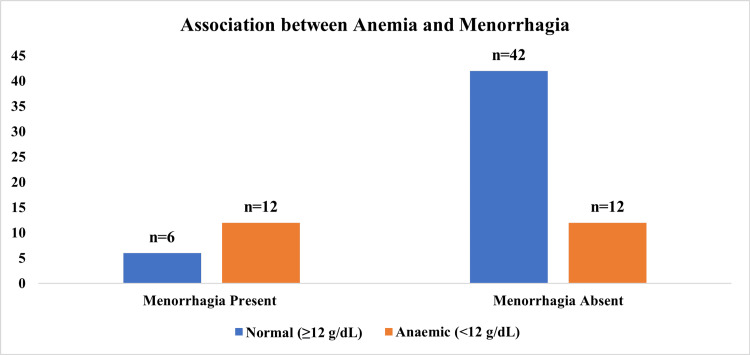
Association between anemia and menorrhagia (N=72) Chi-square test used. p-value <0.05 – Statistically significant.

Figure 3 illustrates the association between anemia and menorrhagia among the study participants. Among the 18 girls with menorrhagia, a higher proportion of girls were anemic (n=12, 66.67%), and this relationship was statistically significant (χ² = 5.9, p < 0.05). The figure illustrates the direct association between excessive menstrual blood loss and reduced hemoglobin levels.

Table 5 shows the practices and awareness of the study participants regarding menstrual hygiene. Most participants (n=60, 83.3%) used sanitary pads, whereas 13.9% (n=10) used cloth. However, 22.2% (n=16) reported changing absorbents ≤ 2 times per day, reflecting inadequate hygiene practice. Clinical correlates of poor hygiene included pallor (n=20, 27.8%), acne (n=18, 25%), and hirsutism (n=8, 11.1%), pointing toward suboptimal awareness of menstrual health and personal hygiene.

**Table 5:** Menstrual hygiene practices and health awareness (N=72)

Practice/Awareness Parameters	Frequency n (%)
Type of Menstrual Absorbent Used	
Sanitary pads	60 (83.3)
Cloth	10 (13.9)
Others	2 (2.8)
Frequency of Absorbent Change per Day	
≤2 times (inadequate)	16 (22.2)
3–4 times (adequate)	42 (58.3)
≥5 times	14 (19.4)
Clinical Findings Related to Hygiene/Nutrition	
Pallor	20 (27.8)
Acne	18 (25.0)
Hirsutism (FG score ≥8)	8 (11.1)

## Discussion

The present hospital-based cross-sectional study among 72 adolescent girls revealed a mean age at menarche of 12.6 ± 1.2 years, aligning with global norms reported in recent literature. Marques et al. in Portugal observed a mean menarcheal age of 12.4 years and identified overweight as an influencing factor for earlier onset [11]. In our study, 25% were overweight or obese, and the prevalence of menstrual irregularity was significantly higher among this group (p = 0.013), confirming that increasing adiposity disrupts hypothalamic-pituitary-ovarian axis function. Similarly, Rigon et al. from Italy reported that obese adolescents had a higher frequency of irregular cycles and dysmenorrhea compared to those with normal BMI [12]. Together, these findings suggest that the trend toward early menarche and metabolic imbalance is increasingly linked to lifestyle transitions in adolescent girls.

A high prevalence of menstrual disorders (94.4%) was identified in our population, of which dysmenorrhea (72.2%) and premenstrual syndrome (75.0%) were most frequent. This concurs with the observations of Varghese et al. in Kerala, who reported dysmenorrhea in 69.3% and premenstrual symptoms in 73% of participants [13]. The predominance of pain-related disorders in both South Indian studies underlines the role of nutritional anemia, stress, and hormonal immaturity in early post-menarchal years. Omidvar et al. conducted a community survey in urban South India and found that 74.3% of adolescents experienced dysmenorrhea, with 25% reporting menorrhagia [14]. Our study’s menorrhagia rate of 25% closely matches these results, confirming the consistency of menstrual morbidity across regional populations.

Menstrual hygiene and awareness directly influence reproductive health outcomes. In the present study, 83.3% used sanitary pads, 13.9% used cloth, and 22.2% reported inadequate absorbent change (<2 times/day), which parallels findings from Deshpande et al. in Maharashtra, where 82.3% used sanitary pads, but hygienic disposal and frequency of change were suboptimal [15]. Such unhygienic practices contribute to local infections and poor school attendance. Our data showed school absenteeism in 38.9% and social restriction in 47.2% of participants, emphasizing the psychosocial dimension of menstrual morbidity.

Expanding from localized patterns to national trends, Chokhandre et al. utilized the National Family Health Survey dataset and reported that 42% of adolescent girls in India experience some form of menstrual disorder, yet only one-fifth sought professional treatment [16]. This aligns with our observation that most participants sought medical advice only after repeated cycles of irregularity or excessive bleeding. Cultural silence and misconceptions remain major barriers. Boruah et al. in Assam found that 58% of adolescents lacked adequate menstrual-hygiene knowledge and only 44% practiced optimal absorbent use [17]. In our study, health awareness was also low; one-fourth of participants demonstrated poor knowledge regarding the physiological basis of menstruation and its disorders, underlining an unmet need for health education even among urban school-attending girls.

Regarding symptom prevalence, the 72.2% dysmenorrhea in our study aligns with the 71% global average reported by Ju et al. in their epidemiological review of dysmenorrhea across 52 studies [18]. They found that moderate-to-severe pain affected daily functioning in nearly half of affected adolescents, consistent with our mean pain symptom score of 8.4 ± 3.2 and WHODAS disability score of 8.6 ± 3.5, indicating measurable functional limitation. The positive correlation between WHODAS and MSQ scores (r = 0.62, p < 0.001) demonstrates the clear impact of menstrual disorders on daily living, corroborating global data that dysmenorrhea is a major cause of school absenteeism and psychological distress.

Digital epidemiology has recently emerged as a novel method for monitoring menstrual health. Symul et al. utilized mobile health applications to analyze over 200,000 menstrual cycles worldwide and found that menstrual irregularity was strongly correlated with BMI and stress, reinforcing our findings [19]. Our study, though limited in sample size, supports the need for community-based digital surveillance systems in India, which can help adolescents track cycle length and detect early deviations from normal patterns. This digital approach is especially relevant in the context of post-pandemic hybrid education, where remote health monitoring can improve early diagnosis.

Mirzaei Salehabadi et al. further provided statistical insights into the recall bias surrounding menarcheal age, proposing biostatistical models to correct under- or over-reporting [20]. Our mean menarcheal age of 12.6 years matches their modeled median of 12.7 years, validating the reliability of our self-reported data. Understanding menarcheal trends remains vital since early menarche increases the risk of metabolic and reproductive disorders later in life.

The menstrual hygiene component of our study showed that 27.8% had pallor and 33.3% were anemic (Hb < 12 g/dL). Comparable findings were reported by El-Gilany et al. in Egypt, where poor menstrual hygiene was significantly associated with anemia and genital infections [21]. This suggests that inadequate hygiene during menstruation has both local and systemic health implications. Likewise, Esimai and Esan in Nigeria found that 31% of college girls reported menstrual abnormalities but lacked awareness of available care services [22]. Our similar patterns of low awareness and anemia emphasize the trans-regional nature of menstrual-health neglect.

From a Southeast Asian perspective, Lee et al. surveyed adolescent girls in Malaysia and reported irregular cycles in 24.7% and dysmenorrhea in 69%, mirroring our findings [23]. They also observed that menstrual discomfort was often normalized, leading to delayed medical consultation, a phenomenon also seen among our participants. The cultural normalization of pain continues to prevent adolescents from perceiving dysmenorrhea as a treatable condition.

Regional comparisons highlight sociocultural influences. Among Lebanese nursing students, Karout et al. documented premenstrual syndrome in 61% and irregular cycles in 30%, with stress and dietary imbalance as contributing factors [24]. In our sample, 75% reported premenstrual symptoms, slightly higher than Lebanese data, possibly due to different assessment tools and dietary patterns. These cross-country parallels underscore that stress, nutritional deficiencies, and hormonal factors contribute universally to adolescent menstrual morbidity.

Latin American data also reinforce the global burden. Pitangui et al. reported dysmenorrhea in 65.8% and daily-activity restriction in 46.7% of Brazilian adolescents [25]. Our corresponding figures, 72.2% dysmenorrhea and 47.2% restricted activity, are remarkably close, confirming that functional impairment is a consistent global outcome of menstrual disorders. Educational absenteeism in our study (38.9%) aligns with Pitangui’s observation of 40-45% school absence, highlighting the educational disadvantage imposed by menstrual morbidity.

Beyond individual health effects, menstrual disorders intersect with Water, Sanitation, and Hygiene (WASH) conditions. Das et al. in Odisha demonstrated that inadequate access to sanitary facilities increased the risk of urogenital infection (adjusted OR = 1.74) [26]. Although our hospital setting ensured access to sanitary products, nearly one-fourth of participants reported changing absorbents less than twice daily, reflecting persisting hygiene gaps. Addressing WASH inequalities is therefore essential for menstrual-health promotion, particularly in rural and low-income urban communities.

Synthesizing evidence from multiple Indian states, van Eijk et al. conducted a meta-analysis and concluded that 48% of Indian adolescent girls lack complete menstrual-hygiene awareness, with irregular cycles affecting one-third [27]. These national statistics resonate with our study’s findings that one-third of participants had anemia and one-eighth demonstrated clinical or ultrasound features of PCOS. The meta-analysis emphasizes that menstrual hygiene management must integrate both education and clinical screening to achieve tangible health gains.

Globally, menstrual hygiene has evolved from a personal issue to a recognized public-health priority. Sommer et al. in the United States defined menstrual hygiene management (MHM) as a core component of health equity, noting that taboos and infrastructure deficits perpetuate gender disparities in education [28]. Our findings, nearly 39% absenteeism and 47% restriction of physical activities, echo this gendered disadvantage, making a strong case for integrating menstrual-health counseling into adolescent-friendly school programs. Furthermore, Long et al. innovatively used participatory games to study menstrual hygiene in rural Bolivia and found that peer-led interactive learning improved knowledge retention and reduced stigma [29]. Such participatory methods could be effectively adopted in Indian schools to normalize menstrual discussions and foster supportive peer environments.

Our study contributes uniquely by combining clinical, biochemical, and psychosocial assessment within a tertiary-care framework-linking hormonal and anthropometric correlates to functional outcomes. The statistical correlation between WHODAS and MSQ scores provides quantitative validation of the subjective disability experienced during menstruation, an area often overlooked in community surveys.

The present study has several notable strengths. The structured use of validated and widely accepted clinical instruments, including the Visual Analogue Scale (VAS), Pictorial Blood Assessment Chart (PBAC), Premenstrual Symptoms Screening Tool (PSST), and WHODAS 2.0, enhances the reliability and clinical credibility of the findings compared to questionnaire-only, school-based surveys. The incorporation of patient-reported outcomes along with clinical examination allows a comprehensive assessment of menstrual disorders and their functional impact. Additionally, the use of brief, standardized tools supports the potential applicability of this approach in routine outpatient and primary care settings. The clinic-based sample drawn from a tertiary care center serving both urban and rural populations also contributed to a socio-demographically diverse cohort.

Despite these strengths, certain limitations must be acknowledged. As a hospital-based cross-sectional study, the sample is subject to selection bias, as adolescents seeking healthcare are more likely to have symptomatic or severe menstrual disorders. This may lead to overestimation of prevalence and severity compared to the general adolescent population, thereby limiting external validity. The relatively small sample size constrains generalizability and reduces statistical power to detect modest associations. Recall bias may have influenced self-reported variables such as age at menarche and menstrual characteristics. Hormonal assays and comprehensive endocrine evaluations were not uniformly performed due to resource constraints, limiting etiological interpretation. Although investigations such as ultrasonography were guided by clinical indications, variability in diagnostic evaluation may have occurred. Finally, the cross-sectional design precludes causal inference. Notwithstanding these limitations, the study provides meaningful baseline data on menstrual disorders and their functional consequences among adolescent girls, underscoring the need for larger, longitudinal, population-based studies.

## Conclusions

This study highlights that menstrual disorders constitute a major but often neglected aspect of adolescent health, with nearly 9 out of 10 girls in this cohort experiencing at least one form of menstrual morbidity. Dysmenorrhea and premenstrual syndrome emerged as the most prevalent conditions, significantly affecting daily functioning, school attendance, and psychosocial well-being. The findings emphasize that nutritional status, body mass index, and polycystic ovarian morphology are key clinical correlates influencing menstrual irregularity. Moreover, the coexistence of anemia, poor hygiene practices, and limited awareness underscores the intertwined nature of biological and sociocultural determinants in menstrual health.

The positive correlation between menstrual symptom severity and disability scores demonstrates that menstrual disorders extend beyond physical discomfort to encompass measurable psychosocial disability. This necessitates a multidimensional approach integrating clinical screening, health education, nutritional support, and menstrual-hygiene promotion within adolescent health programs.

## Appendices

**Table 6:** Questionnaire Scoring and Interpretation: (Total Score Range: 0 to 96) 0–24: Minimal symptoms, 25–48: Mild symptoms, 49–72: Moderate symptoms, >72: Severe symptoms

Section A – Participant Identification			
CRF ID No.: __________			
Date of Recruitment: ___ / ___ / 2025			
Name: _________________________________			
Age: ______ years			
Hospital OP/IP No.: ____________________			
Address: ______________________________			
Contact No.: ___________________________			
Section B – Demographic & Socioeconomic Data (Modified Kuppuswamy Scale 2024)			
Religion: ☐ Hindu ☐ Muslim ☐ Christian ☐ Other			
Education: ☐ Illiterate ☐ Primary ☐ Secondary ☐ Graduate/Postgraduate			
Occupation: ☐ Student ☐ Homemaker ☐ Employed ☐ Other: ______			
Socioeconomic Class: ☐ Upper ☐ Upper Middle ☐ Lower Middle ☐ Upper Lower ☐ Lower			
Section C – Menstrual History (Scales: PBAC, VAS, PSST)			
Age at Menarche: ______ years			
Cycle Length: ______ days			
Duration of Flow: ______ days			
Amount of Bleeding (PBAC Score): ☐ <100 ☐ ≥100			
Regularity: ☐ Regular ☐ Irregular			
Menstrual Symptoms Checklist: ☐ Dysmenorrhea ☐ Menorrhagia ☐ Oligomenorrhea ☐ Polymenorrhea ☐ Amenorrhea ☐ PMS ☐ Others: ______			
Pain Severity (VAS 0–10): _____ / 10			
PMS Severity (PSST Score): ☐ None ☐ Mild ☐ Moderate ☐ Severe			
Section D – Clinical Examination (WHO BMI-for-Age, Ferriman–Gallwey)			
Height: ______ cm Weight: ______ kg BMI: ______ kg/m²			
Pallor: ☐ Present ☐ Absent			
Hirsutism (Ferriman–Gallwey Score): ______			
Acne: ☐ Present ☐ Absent			
Thyroid Enlargement: ☐ Present ☐ Absent			
Section E – Investigations (WHO Anaemia Classification)			
Haemoglobin: ______ g/dL ☐ None ☐ Mild ☐ Moderate ☐ Severe			
Thyroid Profile: ☐ Normal ☐ Abnormal			
Pelvic Ultrasound: ☐ Normal ☐ PCOS features ☐ Other: ______			
Other Tests: ___________________________			
Section F – Impact on Daily Life (WHODAS 2.0 Short Form)			
School Absenteeism: ☐ Yes ☐ No If yes, days missed: ______			
Restriction of Social/Physical Activities (WHODAS Score): ______			
Menstrual Hygiene: • Absorbent Used: ☐ Pads ☐ Cloth ☐ Others • Frequency of Change: ______ per day			
Section G – Final Classification of Menstrual Disorder			
☐ Dysmenorrhea ☐ Menorrhagia ☐ Oligomenorrhea ☐ Polymenorrhea ☐ Amenorrhea ☐ Irregular Cycles ☐ Other: ______			
Section H – Menstrual Symptom Questionnaire (MSQ) (Validated Tool)			
Instructions: Please mark the severity of each symptom during your last three menstrual cycles.			
Scale: 0 = Not at all | 1 = Mild | 2 = Moderate | 3 = Severe | 4 = Very Severe			
S.No	Symptom		Score
Pain Symptoms			
1	Lower abdominal cramps	☐ 0 ☐ 1 ☐ 2 ☐ 3 ☐ 4	
2	Backache	☐ 0 ☐ 1 ☐ 2 ☐ 3 ☐ 4	
3	Headache	☐ 0 ☐ 1 ☐ 2 ☐ 3 ☐ 4	
4	Breast tenderness	☐ 0 ☐ 1 ☐ 2 ☐ 3 ☐ 4	
Autonomic Symptoms			
5	Dizziness / lightheadedness	☐ 0 ☐ 1 ☐ 2 ☐ 3 ☐ 4	
6	Nausea / vomiting	☐ 0 ☐ 1 ☐ 2 ☐ 3 ☐ 4	
7	Fatigue/tiredness	☐ 0 ☐ 1 ☐ 2 ☐ 3 ☐ 4	
Psychological Symptoms			
8	Irritability	☐ 0 ☐ 1 ☐ 2 ☐ 3 ☐ 4	
9	Mood swings	☐ 0 ☐ 1 ☐ 2 ☐ 3 ☐ 4	
10	Anxiety / nervousness	☐ 0 ☐ 1 ☐ 2 ☐ 3 ☐ 4	
11	Difficulty concentrating	☐ 0 ☐ 1 ☐ 2 ☐ 3 ☐ 4	
Behavioral & Lifestyle Impact			
12	Reduced school/work performance	☐ 0 ☐ 1 ☐ 2 ☐ 3 ☐ 4	
13	Social withdrawal	☐ 0 ☐ 1 ☐ 2 ☐ 3 ☐ 4	
14	Sleep disturbances	☐ 0 ☐ 1 ☐ 2 ☐ 3 ☐ 4	
15	Decreased appetite	☐ 0 ☐ 1 ☐ 2 ☐ 3 ☐ 4	
Cycle & Flow Patterns			
16	Irregular cycles	☐ 0 ☐ 1 ☐ 2 ☐ 3 ☐ 4	
17	Heavy flow	☐ 0 ☐ 1 ☐ 2 ☐ 3 ☐ 4	
18	Passage of clots	☐ 0 ☐ 1 ☐ 2 ☐ 3 ☐ 4	
19	Short cycles (<21 days)	☐ 0 ☐ 1 ☐ 2 ☐ 3 ☐ 4	
20	Prolonged cycles (>35 days)	☐ 0 ☐ 1 ☐ 2 ☐ 3 ☐ 4	
Other Common Symptoms			
21	Lower limb swelling	☐ 0 ☐ 1 ☐ 2 ☐ 3 ☐ 4	
22	Acne flare-up	☐ 0 ☐ 1 ☐ 2 ☐ 3 ☐ 4	
23	Excessive hair growth	☐ 0 ☐ 1 ☐ 2 ☐ 3 ☐ 4	
24	Weight changes	☐ 0 ☐ 1 ☐ 2 ☐ 3 ☐ 4	
